# Enhancement of Photoluminescence from Semiconducting Nanotubes in Aqueous Suspensions due to Cysteine and Dithiothreitol Doping: Influence of the Sonication Treatment

**DOI:** 10.1186/s11671-016-1708-y

**Published:** 2016-11-08

**Authors:** Nikita V. Kurnosov, Victor S. Leontiev, Victor A. Karachevtsev

**Affiliations:** B.I. Verkin Institute for Low Temperature Physics and Engineering, National Academy of Sciences of Ukraine, 47 Nauky Ave, 61103 Kharkov, Ukraine

**Keywords:** Nanotubes, DNA, Cysteine, Dithiothreitol, Photoluminescence spectroscopy

## Abstract

The influence of tip sonication duration on the spectral characteristics of carbon single-walled nanotubes (SWNTs) in aqueous suspension with single-stranded DNA (ssDNA) has been studied by NIR luminescence, NIR absorption, and Raman spectroscopy. It was revealed that prolongation of sonication leads to weakening of the SWNT polymer coverage and appearance of additional defects on the nanotube surface. Prolongation of the tip sonication treatment of SWNT/ssDNA from 30 to 90 min leads to the increase of the number of individual nanotubes in the aqueous suspension, but it significantly decreases the photoluminescence (PL) from semiconducting SWNTs because more defects are formed on the nanotube surface. At probing the SWNT/ssDNA emission with cysteine or dithiothreitol (DTT) doping the nanotube aqueous suspension showed the different PL intensity enhancement depending on the duration of the sonication treatment and on the ability of these reducing agents to passivate emission-quenching defects on the carbon nanotube sidewall. The magnitude of the PL enhancement rises with sonication prolongation and depends on the nanotube chirality. Tight and ordered polymer coverage of (6,4) nanotubes hampers the access of the reducing agent to emission-quenching defects on the nanotube surface and provides the weaker PL intensity increasing while (7,5) nanotubes show the strongest reaction to the doping effect. The comparison of cysteine and DTT ability to passivate the emission-quenching defects showed the higher efficiency of DTT doping. This prevailing is explained by the stronger reducing activity of DTT which is determined by a lower redox potential of this molecule.

## Background

The photoluminescence (PL) from single-walled carbon nanotubes (SWNTs) is characterized by a very high sensitivity to environmental/external influence, and therefore, this emission can be applied to various fields including bioimaging and sensing [1]. PL originates from semiconducting SWNTs and locates in the near-infrared (NIR) spectral region [2].

PL from SWNTs is caused by excitons [3–5]. The energy of Coulombic interaction between components of an electron-hole pair in semiconducting nanotube is in the order of 400 meV [3–5]. Such huge binding energy provides the high exciton mobility at room temperature with the large diffusion length (more than 120 nm) [6–9]. The high exciton mobility provides both advantages and disadvantages; namely, the high sensitivity of PL towards external influence allows detection of single molecules [10]. But excitons in SWNTs are also very sensitive to the nanotube defects, and this leads to decreased quantum yield [11]. PL-quenching defects can appear in the aqueous environment due to the presence of dissolved oxygen [12]. It was shown that the use of small reducing molecules, namely dithiothreitol (DTT), β-mercaptoethanol (BME), and Trolox can passivate the action of defects and quantum yield of nanotube PL is increased/restored [11].

Nanotubes emit in aqueous environment only if they are individualized [2] or in small bundles containing only semiconducting nanotubes. For this purpose, different surfactants or polymers are often exploited. Among polymers such biopolymer as DNA (both oligonucleotides and long strands) is effective due to wrapping around nanotubes [13]. The coverage of the SWNT surface clearly influences observed PL band parameters (mainly intensity and spectral position) [14, 15]. Further, at the investigation of reducing effect on the PL from SWNTs, it was suggested that PL change at addition of reducing molecules to SWNTs is greatly affected by the adsorbed polymer [16, 17]. Also, the addition of photoluminescence-restoring molecules can be useful not only for increase of the PL intensity and sensing applications but also can serve as an indirect emission probe of the polymer coverage of SWNTs [17].

Ultrasonication is the widespread technique used to obtain individual SWNTs [18]. Tip sonication and sonication through water/oil bath are two mostly exploited sonication methods. The main purpose of the sonication treatment lies in splitting of nanotube bundles to allow further functionalization with various molecules including DNA [19, 20]. But this treatment also influences on individual nanotubes leading to damage [18] (appearance of defects which are observed in Raman spectra) and scission [21]. Nevertheless, the sonication allows preparation of aqueous suspension with high content of individualized SWNTs necessary for observation of the SWNT emission and NIR absorption. It was shown that time of sonication affects greatly content of individual SWNTs (controlled by absorbance) and their damage (controlled by ratio in Raman spectra between defect-induced D band and tangential G^+^ band, denoted as D/G^+^) [19].

Sonication has an influence on DNA too. It lies mainly in strand fragmentation [22] and also can lead to disruption of bonds between strands in double-stranded DNA [23]. It is also known that length of DNA fragments greatly affects their adsorption onto SWNT surface. So, sonication will affect the observed PL from nanotubes in two ways: through influence on SWNTs directly and through influence on DNA to be adsorbed. The process of the DNA adsorption can have an effect on the relative content of SWNTs in the suspension and their surface coverage.

Thiol compounds usually possess redox activity due to the presence of reactive thiol (–SH) group. In the pioneering work concerning the influence of reducing agents on the SWNT PL [11], the two used compounds (BME and DTT) were thiols. Such important biological thiol as cysteine fulfills its biological functions due to redox activity [24, 25], and this fact can also be used for its detection [26, 27]. Cysteine was proven to have qualitatively similar to DTT (enhancing) effect on the SWNT emission [28], and some external factors (tip or bath sonication) affect the PL intensity-concentration dependence [29].

In the present work, we have investigated the influence of tip sonication duration on the spectral characteristics of DNA-wrapped SWNTs in aqueous suspension controlling the PL enhancement as a result of cysteine or DTT addition. Such spectral parameters of PL bands as the intensity, the spectral position and the spectral width were analyzed before and after cysteine addition. The PL and NIR absorption spectra of undoped suspensions were analyzed too. Raman spectroscopy was exploited to control the defect appearance as a result of prolongation of sonication treatment. We have also compared the influence of cysteine and DTT on the PL, observed for the same SWNTs:ssDNA suspensions.

## Methods

### Preparation of SWNTs:ssDNA Aqueous Suspensions

SWNTs used in the experiments were produced by CoMoCat method [30] (SouthWest NanoTechnologies, USA). Semiconducting SWNTs with (6,5) chirality (SWNT® SG 65) prevailed in the starting material. Single-stranded DNA (obtained from the native, extracted from chicken erythrocytes double-stranded DNA [31]) dissolved in 0.005 M Na^+^ cacodylate buffer (pH 7) (Serva, Germany) with 0.005 M NaCl was used for the preparation of SWNT aqueous suspensions. Steady SWNT aqueous suspensions were prepared through sonication of nanotube bundles in solution with the biopolymer. Two different suspensions with SWNTs:ssDNA 1:1 initial weight ratio were prepared using tip method with different total time of sonication: 30 and 90 min. All other parameters of sonication were the same (8 W, 22 kHz). As a result of the sonication treatment, ssDNA was fragmented [23]. Ultracentrifugation (70,000*g*, 60 min) followed the sonication treatments.

### Titration

Stock aqueous solutions of cysteine and DTT at concentrations ranging from 2 × 10^−7^ to 10^−1^ M were prepared before a titration of SWNT suspensions. In titration experiments, 2 μL of 2 × 10^−7^ M cysteine solution was the minimal dose added into the nanotube suspension portion (400 μL). The cysteine concentration in the suspension varied from 10^−9^ to 10^−3^ M. Spectroscopic measurements followed after up to 5-min delay required to each of the thermodynamic equilibrium. For each of two suspensions studied exactly the same titration procedure was performed for cysteine and DTT.

### Spectroscopic Measurements

PL from semiconducting carbon nanotubes was analyzed using a NIR spectrometer with the signal detection by a thermocooled CCD camera. Emission was excited with a diode-pumped solid-state (DPSS) green laser (*λ*_exc_ = 532 nm (2.33 eV), 5 mW). Laser excitation power was checked before and after registration of each spectrum.

The absorption spectra of nanotube suspensions were obtained using NIR spectrometer equipped with thermocooled InGaAs photodiode (900–1600 nm). Obtained spectra were combined with those observed using Hitachi M 356 spectrophotometer (in the spectral region 360–1150 nm). NIR spectrometers were calibrated with Ne lamp spectrum before and after measurements. Quartz cuvettes with 2-mm path length were used in absorption experiments.

Raman scattering of the nanotube suspensions was excited with argon gas laser (*λ*_exc_ = 488 nm, 75 mW), and spectra were analyzed using double-grating monochromator and detected with a thermocooled CCD camera. Spectra were obtained in the spectral region 1100–1800 cm^−1^ in which both G and D bands of SWNTs are located.

## Results and Discussion

**Fig. 1:**
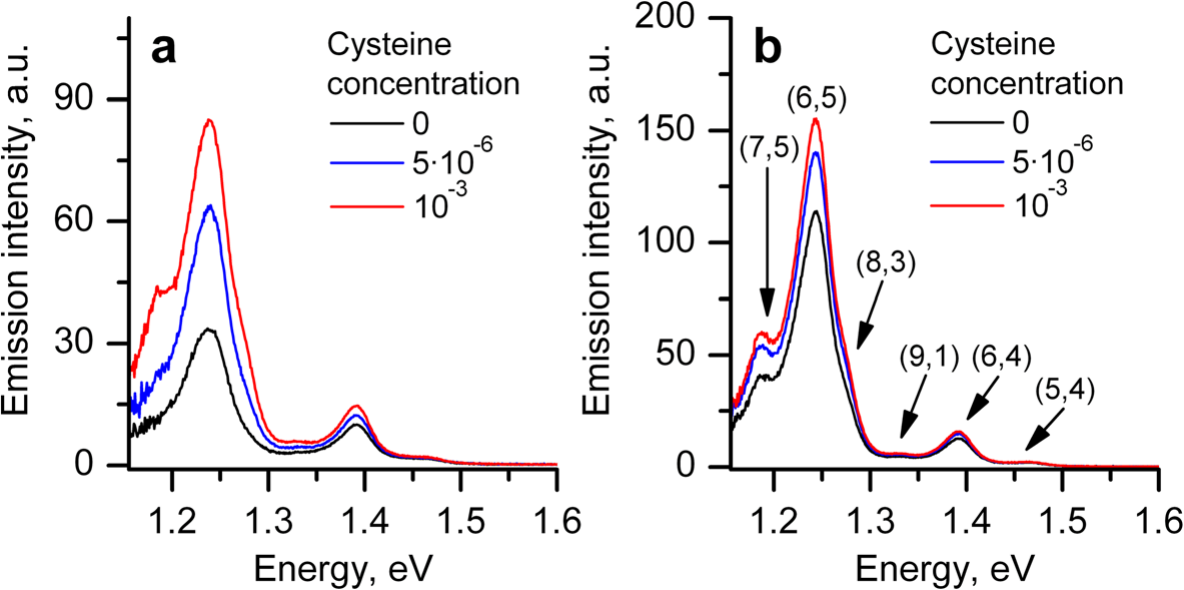
Photoluminescence spectra of nanotube aqueous suspensions with ssDNA prepared with 90-min (**a**) and 30-min (**b**) tip sonication at different cysteine concentrations

The PL spectra of two SWNTs:ssDNA suspensions prepared by sonication treatment during 30 and 90 min (further denoted as SWNTs:ssDNA 30’ and SWNTs:ssDNA 90’) at different concentrations of cysteine are presented in Fig. 1.

In the PL spectrum, several bands are observed, which can be assigned to a certain nanotube chirality [32] indicated in Fig. 1b. The two most intensive bands correspond to (7,5) and (6,5) nanotubes with spectral positions (for SWNTs:ssDNA 30’ suspension) at 1.186 and 1.242 eV, respectively. Note also a noticeable band assigned to (6,4) nanotubes located at 1.391 eV. Among them, the emission from (6,5) nanotubes is prevailing (more than 50 %). It follows from Fig. 1 that the rise of the cysteine concentration in suspensions was accompanied by the increase of the nanotube PL intensity. It indicates that the PL intensity enhancement at the final cysteine concentration (10^−3^ M) is much higher in case of 90-min sonicated suspension. The ratios of (6,5) band intensities before and after cysteine doping (at 10^−3^ M) are 2.5 and 1.37 for SWNTs:ssDNA 90’ and SWNTs:ssDNA 30’ suspensions, respectively. The spectral analysis shows that for each separate suspension, the intensity enhancement depends on the SWNT chirality. The values of integral intensity increase for bands (7,5), (6,5), and (6,4) follow the order (7,5) > (6,5) > (6,4): 3.63, 2.48, 1.39 (SWNTs:ssDNA 90’) and 1.39, 1.36, 1.22 (SWNTs:ssDNA 30’). All these values were obtained from *I*_*S*_/*I*_0_ ratio, where *I*_0_ and *I*_*S*_ denote the integral intensity before and after titration. *I*_*S*_ value was taken when the concentration curve acquires a saturation or semi-saturation, and in this case, *I*_*S*_ was practically determined at final concentration of added compound. Note that weak intensity bands assigned to (9,1) and (5,4) nanotubes have even smaller effect of the intensity increase at long sonication than that of (6,4) band.

**Fig. 2:**
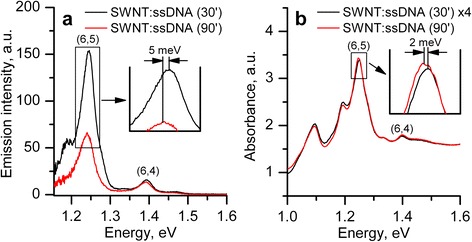
**a** Photoluminescence spectra of undoped SWNTs:ssDNA suspensions prepared with sonication for 30 (*black line*) and 90 (*red line*) min. The spectra are corrected for re-absorption as described in the text. **b** NIR absorption spectra of undoped SWNTs:ssDNA suspensions

We compared the PL and NIR absorption spectra of undoped suspensions exposed to sonication for different times (Fig. 2). To take into account the PL re-absorption by nanotubes placed between the laser track and cuvette wall, we obtained SWNT PL spectra at the two distances between laser track and cuvette wall (0.5 and 1 mm) and calculated the PL spectra without re-absorption of the emission (Fig. 2a). The NIR absorption spectra of two undoped suspensions are presented in Fig. 2b (to compare these spectra properly, the spectrum of suspension sonicated for 30 min was multiplied by factor of 4). The insets in both Fig. 2a, b show the spectral shift of the most intensive band corresponding to (6,5) nanotubes observed in two spectra. In the PL spectrum, the bands corresponding to (6,5) and (7,5) nanotubes after 90-min sonication are red-shifted by 5 and 6 meV, respectively, in comparison with short sonication. In the NIR absorption spectra, this spectral shift is smaller (~2 meV). Note that the PL band assigned to (7,5) nanotubes in the suspension sonicated for 90 min is weaker and does not appear as a distinct shoulder on the slope of the intense (6,5) band as it is observed in the suspension prepared by short-time sonication. The similar shift of another well-resolved (6,4) band is also observed but it is noticeably smaller, not above 1.5 meV in PL spectra and less than 1 meV in the NIR absorption spectrum. The observed red shift of the bands in the emission and absorption spectra of nanotubes indicates that longer sonication alters the surface polymer coating of nanotubes and as a result of this treatment, the access of water molecules to the nanotube surface is expanded.

**Fig. 3:**
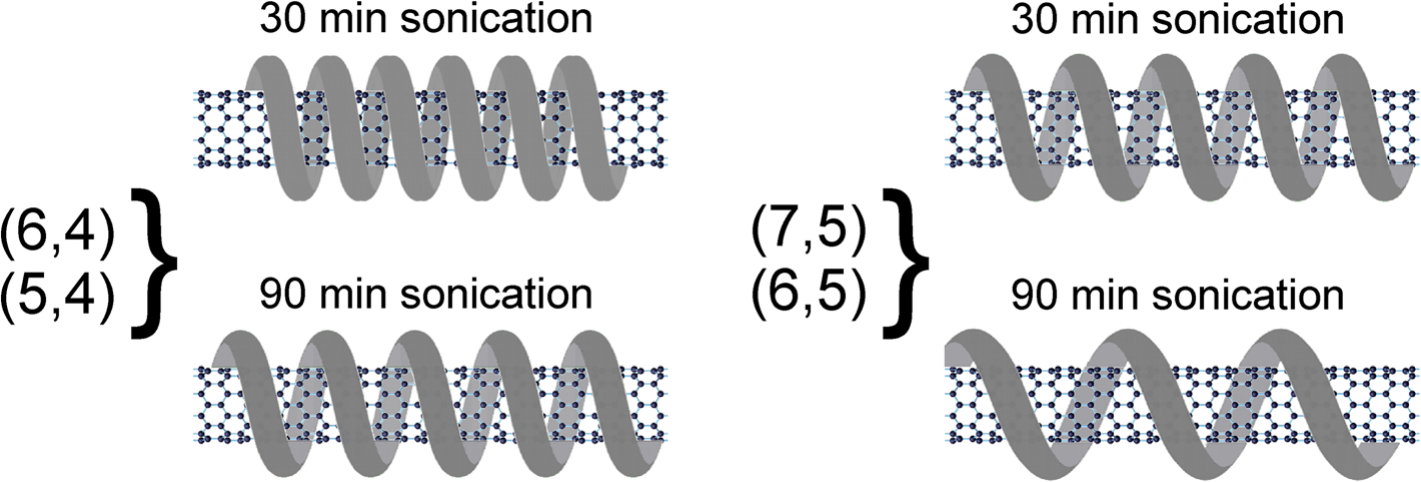
Schematic picture showing the polymer wrapping around the (6,4) and (5,4) nanotubes with small pitch (*left*, *top*), which increases slightly after 90-min sonication (*left*, *bottom*); while for (7,5) and (6,5) nanotubes, the pitch enlarges significantly after prolonged sonication (*right*, *bottom*)

These experimental observations point out that DNA adsorbed on the surface of (7,5) or (6,5) nanotubes at long sonication does not provide the tight coverage. It is possible that wrapped DNA has a larger pitch as it is schematically shown in Fig. 3 (right). At that H-bonding between neighboring coils becomes impossible and reliability of the polymer two-dimensional sheet (DNA *β*-barrel structure [33]) preventing water molecules is lost. In the *β*-barrel structure model, the backbone and bases of ssDNA are arranged helically on an imaginary cylinder with the hollow interior of the structure which permits the insertion of SWNT of a certain diameter. The molecular dynamics simulation showed that under mutual recognition of (6,5) SWNT by sequence (TAT)_4_, DNA forms an ordered right-handed helically wrapped barrel, stabilized by intra-strand and inter-strand hydrogen bonding [34]. The same sequence on the larger diameter (8,7), SWNT forms a much more disordered structure than that on (6,5) nanotube. These simulations revealed that the DNA sequence-specific binding strength correlates with selectivity to carbon nanotube. For example, it was shown that the sequence (TAT)_4_ which recognizes (6,5) nanotubes binds ~20 times stronger than either (TAT)_3_TA or (TAT)_4_T [34]. Note that the formation of a highly ordered oligomer arrangement on SWNT is considered to explain the recognition ability of certain sequence of DNA [35]. In our case, we suggest that ssDNA binds stronger with (6,4) nanotubes (in spite of arbitrary sequence of bases in ssDNA) than with (6,5) and (7,5) ones. This conclusion is based on the small red shift of (6,4) band after prolonged sonication and weaker response to cysteine doping (Fig. 1). Less dense polymer sheet on the nanotube allows the adsorption of cysteine molecules on the SWNT surface between the coils with big pitch, including the adsorption on the defect sites.

Another conclusion is followed from the analysis of spectra presented in Fig. 2 that a longer sonication of SWNT/ssDNA (90 min) leads to increase of the number of individual nanotubes in an aqueous suspension by about four times, but this treatment significantly quenches the emission (greater than eightfold). The main reason of this observation lies in the enhancement of the number of defects on the nanotube surface that quench the emission.

Taking into account the re-absorption in PL spectra also allowed us to consider the relative integral intensities of PL bands (note that re-absorption is especially noticeable when *I*_(6,5)_/*I*_(6,4)_ ratio was estimated). In our compatible estimations for normalization of integral intensities of different bands, we have chosen the (6,4) band because it is well-resolved and affected very little by longer sonication. So, in the PL spectrum of SWNTs:ssDNA 30’ suspension (presented in Fig. 2a), the relative integral intensities of bands (7,5), (6,5), and (6,4) are rated as 4:10.9:1 while for the SWNTs:ssDNA 90’ suspension, this ratio is drastically different (1.26:5.75:1). Note that in NIR absorption spectra of both undoped suspensions (Fig. 2b), the ratio of intensities of the (7,5), (6,5), and (6,4) bands are practically the same: 5.3:15.4:1 and 4.3:14.2:1 for suspensions SWNTs:ssDNA 30’ and 90’, respectively. These ratios indicate that the longer sonication leads to significantly lower quantum yield of the emission for nanotubes (7,5) and (6,5), and this emission quenching is dependent on the nanotube chirality.

It was revealed that prolongation of sonication leads to weakening of the polymer coverage of SWNTs and appearance of additional defects on the nanotube surface. Earlier, it was shown that absorbance of individual SWNTs in suspension is changed almost linearly with sonication time while for the ratio between defect-induced band and tangential band (D/G^+^ ratio), there was some trend for saturation [19]. In our Raman measurements, we have obtained Raman spectra of undoped suspensions prepared with 30- and 90-min sonication time. Excitation was performed with Ar^+^ (488 nm), so that resonance conditions are fulfilled for both semiconducting and metallic nanotubes. The comparison of spectra normalized to G^+^ band integral intensity showed that longer sonication leads to higher integral intensity of D band (by ~9 %). This is a small increase of the defect number but similar to that observed earlier when different sonication methods were applied [29].

**Fig. 4:**
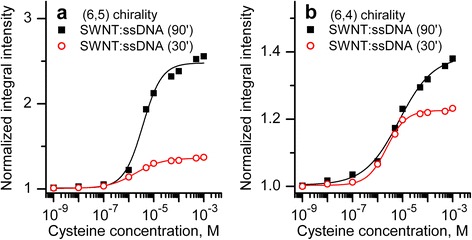
Dependencies of normalized integral intensities of PL bands corresponding to nanotube chirality (6,5) (**a**) and (6,4) (**b**) in SWNTs:ssDNA suspensions sonicated for 90 and 30 min

In the following experiments, we have compared the influence of cysteine doping on the integral intensity of (6,5) and (6,4) PL bands in two suspensions. The obtained dependencies of normalized integral intensities on the cysteine concentration plotted in semilogarithmic scale are shown in Fig. 4.

It follows from Fig. 4b that (6,4) band has similar dependencies of the integral intensity on the cysteine concentration obtained for two suspensions. So, the integral intensity of this band increases by 39 and 22 % for SWNT:ssDNA 90’ and SWNTs:ssDNA 30’ suspensions, respectively, at 10^−3^ M of cysteine concentration. As for the (6,5) band (the intensity and spectral position are greatly different for two undoped suspensions (Fig. 2)), cysteine addition leads to the essential influence on the integral intensity in the SWNTs:ssDNA 90’ suspension. It increases by 148 % after doping while only by 36 % for SWNTs:ssDNA 30’ suspension. So, analyzing results presented in Figs. 2 and 4, we observe some correlation between parameters of PL bands in undoped suspension and their changes at the cysteine addition.

As for the impact of different time of sonication, we assume that similarities (for the (6,4) band) and differences (for (6,5) one) are mainly due to the polymer coverage of corresponding nanotubes. At the qualitative level, the less tight/ordered coverage of nanotube surface with DNA leads to (a) a spectral red shift of the corresponding PL band, the lower initial emission intensity and (b) greater effect of the cysteine addition. This is observed for the (6,5) nanotubes in SWNTs:ssDNA aqueous suspension prepared by long sonication (90 min). We believe that a longer sonication increases the area of the nanotube surface free of polymer due to withdrawing of the part of weakly bound polymer from this surface and making shorter the polymer in suspension. The longer sonication is accompanied with the spectral red shift of the PL band as a result of access of water molecules to the nanotube surface and with lower emission intensity, because SWNT surface is more exposed to dissolved oxygen that facilitates appearance of defects quenching the PL [11, 12]. On the other hand, in this case, SWNT surface defects are more open to reducing agents, which, in turn, cause the PL enhancement [11].

**Fig. 5:**
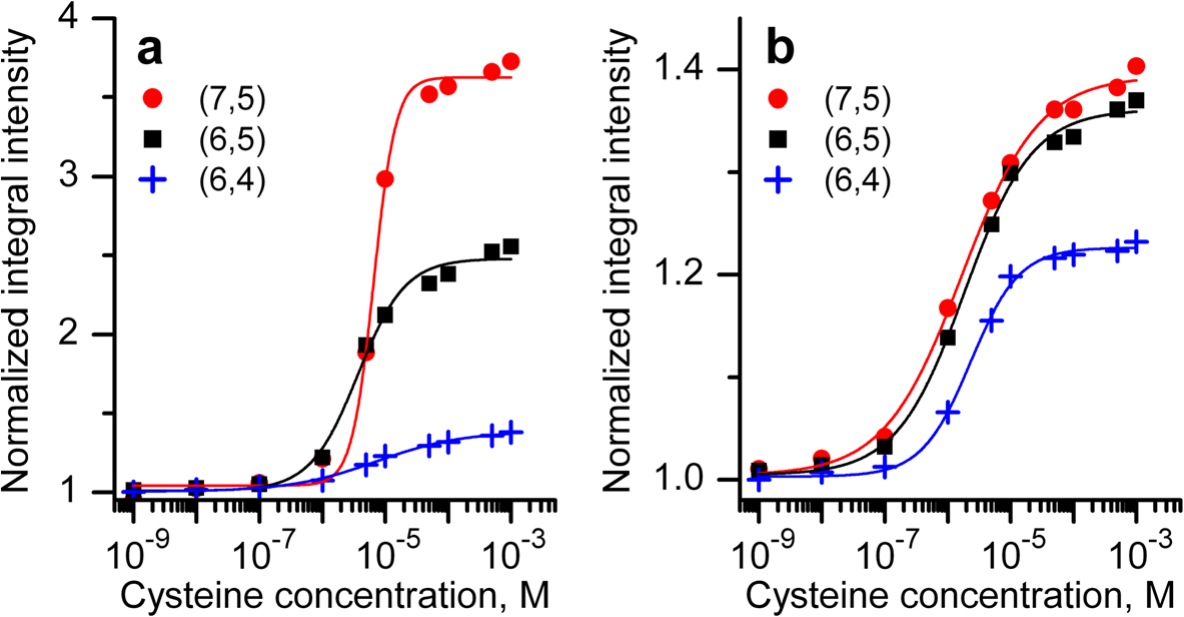
Emission intensity-concentration dependencies for bands (7,5), (6,5), (6,4) observed in spectra of SWNTs:ssDNA 90’ (**a**) and SWNTs:ssDNA 30’ (**b**) suspensions at addition of cysteine

The cysteine addition affects only the intensity of all PL bands but not the spectral position (Fig. 1). Spectral width does not change practically too. From all bands, we observe that the only one exception is the (7,5) band which became narrower at cysteine addition into the SWNTs:ssDNA 90’ suspension (initial full width at half-maximum (FWHM) ~50 meV, final ~40 meV). We assume that this narrowing can appear due to more pronounced action of cysteine on those (7,5) nanotubes with weakly adsorbed ssDNA. Note that despite this narrowing, (7,5) band showed the greatest increase of integral intensity among the observed bands. The integral intensity-concentration dependencies of all analyzed PL bands ((7,5), (6,5), and (6,4)) for each of SWNTs:ssDNA suspensions separately are presented in Fig. 5.

The values of the PL intensity increase for all nanotubes indicated above. The greater discrepancy between concentration dependencies for different chiralities is clearly observed for the SWNT/ssDNA 90’ suspension. We also attribute this to the influence of the ultrasound on DNA and on the polymer adsorption on the surface of different nanotube species, namely longer sonication leads to more unordered polymer coverage. It should be noted that such titration of SWNT suspensions with cysteine (or other reducing agent) with simultaneous control of the PL intensity can serve as an indirect probing of the surface coverage.

**Fig. 6:**
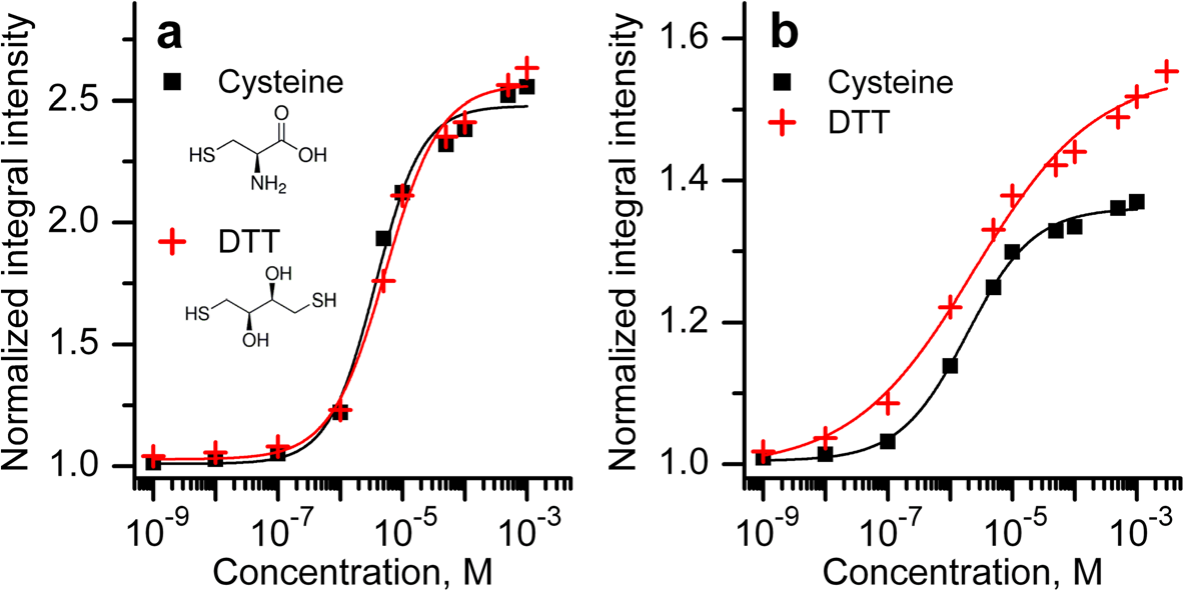
Dependencies of the (6,5) nanotube PL band integral intensity on the concentration of cysteine and DTT obtained for SWNTs:ssDNA suspensions sonicated for 90 (**a**) and 30 (**b**) min

Also, we have titrated both SWNTs:ssDNA 90’ and SWNTs:ssDNA 30’ suspensions with such reducing agent as DTT, which has demonstrated the large PL enhancement earlier [11, 17]. We kept on the same titration procedure used for cysteine (the concentration in suspension ranged from 10^−9^ to 10^−3^ M). The dependencies of the emission of the most intensive band (6,5) on cysteine and DTT concentrations for two suspensions are presented in Fig. 6.

The structures of cysteine and DTT are also shown in Fig. 6a. It follows from obtained dependencies that DTT yields the greater enhancement of the emission than cysteine for both SWNT suspensions. In the SWNTs:ssDNA 90’ suspension, this prevailing is noticeable only slightly; however, for other suspension, this difference is remarkable. In addition, the PL increase starts from the lower concentration (10^−8^ M) in case of the nanotube suspension sonicated for 30 min. In Table 1, the values of the PL enhancement at DTT addition for analyzed nanotube chiralities (6,5) and (6,4) are compared to those obtained for cysteine doping at maximal concentration (10^−3^ M).

**Table 1:** PL increase values (given as (*I*_*S*_/*I*_0_−1) × 100 %) for titration with cysteine and DTT at 10^−3^ M

Nanotube chirality	Sonication (min)	Cysteine (%)	DTT (%)
(6,5)	30	+36	+55
	90	+148	+156
(6,4)	30	+22	+30
	90	+39	+42

Note that DTT yields the higher relative increase of the PL comparing to cysteine for nanotubes of both (6,5) and (6,4) chiralities (Table 1). Higher effectiveness of DTT can appear due to different reducing ability of this compound. In case of the suspension sonicated for 30 min (more tight/uniform polymer coverage of the surface of all SWNTs), greater impact of DTT can be also caused by the fact that at oxidation, one DTT molecule forms intermolecular S–S bond, while two cysteines are usually needed to form dimer at the redox reaction. The reducing activity of DTT or cysteine is quantitatively determined by redox potential of the molecule. The redox potential reflects the ability of the compound to gain or lose electrons at redox reactions. For DTT, this potential is much lower (−0.33 V [36, 37]) comparing to cysteine (−0.22 V [38]) meaning that DTT is a stronger reducer.

## Conclusions

A prolongation of the tip sonication treatment of SWNTs:ssDNA from 30 to 90 min leads to increase of the number of individual nanotubes in aqueous suspension by about four times, but it significantly decreases the emission (eightfold) because of the increased number of defects on the nanotube surface. The longer sonication alters the surface polymer coating of nanotubes, and as a result of this treatment, the access of water molecules to the nanotube surface is expanded. It is accompanied with the red shift of the bands in the emission and absorption spectra of nanotubes.

The strength of the polymer coverage of the nanotube surface and polymer resistance to the sonication depends on the chirality of the nanotubes. The weaker spectral transformation of (6,4) nanotube band can be explained by more ordered polymer adsorption on this nanotube when polymer wraps tightly around the nanotube and restricts the access of water molecules to its surface.

Cysteine or dithiothreitol doping of the nanotube aqueous suspension enhances the PL intensity through the passivation of p-defects on the carbon nanotube sidewall. Note that the magnitude of this enhancement rises with sonication time increasing and depends on the nanotube chirality. The tight and ordered polymer coverage of (6,4) nanotube hampers the access of reducing agent to emission-quenching defects on the nanotube surface and provides the weaker nanotube intensity increasing while (7,5) nanotubes show the strongest reaction to the doping.

A comparison of cysteine and dithiothreitol ability to reduce the emission-quenching defects showed the higher efficiency of DTT doping of the nanotube aqueous suspension. It can be explained by the larger quantity of the thiol groups in DTT (two) and only one group in cysteine structure, leading to stronger reducing activity of DTT displayed by lower redox potential. The prevailing of DTT is more noticeable at 30-min sonication treatment while at 90 min difference between cysteine and DTT is weakened. We assume that at short sonication, there is a significant influence of the polymer coverage of the nanotube surface limiting the access of reducing agents to the quenching defects. At prolonged exposure, the role of the polymer coverage of the nanotube surface as a barrier for the reducing agent is diminished because of the appearance of the significant number of emission-quenching defects which are formed on the nanotube surface free of polymer.

## Abbreviations

DTT: Dithiothreitol
NIR: Near infrared
PL: Photoluminescence
ssDNA: Single-stranded DNA
SWNT: Single-walled carbon nanotube
